# DINO-EYE: self-supervised learning for identification of different optic disc phenotypes in primary open angle glaucoma

**DOI:** 10.1038/s41598-025-33140-1

**Published:** 2026-01-10

**Authors:** Lourdes Grassi, Zhe Fei, Esteban Morales, Joseph Caprioli

**Affiliations:** 1 Glaucoma Division, Ophthalmology, Jules Stein Eye Institute, University of California Los Angeles (UCLA), Los Angeles, CA 90095 USA; 2 Charles Centro Oftalmológico, Buenos Aires, Argentina; 3 Department of Statistics, UC Riverside, Riverside, CA 92521 USA

**Keywords:** Primary open-angle glaucoma, Self-supervised learning, Optic disc photograph, Vision transformer, Fundus photography, Computational biology and bioinformatics, Diseases, Medical research

## Abstract

To develop a self-supervised learning (SSL) model that classifies optic disc phenotypes in primary open angle glaucoma (POAG) and explores novel phenotypic patterns with optic disc photographs (ODPs). We collected 850 ODPs from patients with POAG and applied data augmentation to address class imbalances, yielding 10,493 images. Using the DINO Vision Transformer as the backbone model, we trained an SSL model to extract 2048-dimensional latent features. These features were used for both supervised classification of six known phenotypes and unsupervised clustering. Classification performance was evaluated with Random Forest and XGBoost models. UMAP (Uniform Manifold Approximation and Projection) was used for dimensionality reduction and feature visualization, and attention maps were generated for model interpretability. The DINO-EYE model features enabled phenotype classification with 91% accuracy with Random Forest and 92.1% after merging clinically similar phenotypes. Unsupervised clustering revealed coherent groupings, particularly for concentric thinning and extensive Peripapillary Atrophy (PPA), though no new phenotypes were unanimously confirmed by clinicians. The proposed model outperformed the RETFound SSL model in phenotype classification and demonstrated interpretable attention regions consistent with expert criteria. Our DINO-EYE effectively extracts clinically meaningful features from fundus images and enables accurate classification of optic disc phenotypes in POAG. It surpasses existing SSL models in performance and interpretability, offering promise for real-world glaucoma decision support and individualized care planning.

**Supplementary Information:**

The online version contains supplementary material available at 10.1038/s41598-025-33140-1.

## Introduction

Glaucoma is a chronic progressive optic neuropathy characterized by damage to the optic nerve head and is a leading cause of irreversible blindness worldwide^1^. A recent meta-analysis predicted that by 2040, glaucoma will impact around 111.8 million people^2^. Early detection is a critical component of treatment and disease progression prevention.

Various diagnostic tools are used for glaucoma detection, including Optic Disc Photographs (ODPs), a traditional and relatively inexpensive option for the screening and monitoring of patients. The employment of ODPs in clinical settings led to the classification of Primary Open Angle Glaucoma (POAG) according to the optic disc appearance or phenotype^3^. A recent classification based on phenotypic expression includes: generalized thinning, focal thinning, acquired pit of the optic nerve (APON), tilted, extensive peripapillary atrophy, and broad thinning^4^.

These phenotypes are associated with specific demographics as well as clinical and ophthalmological characteristics, helping to individualize treatment^5^. Although this phenotypic classification can be applied generally, agreement among clinicians proves to be challenging due to interpersonal disagreements, biases, differing personal experiences, and levels of training. Grassi et al.^4^ previously reported a 40% disagreement rate between two graders, and even when a third grader was introduced as a tiebreaker, there remained a 30% rate of disagreement among all three experts.

With the rapid emergence of deep learning, particularly self-supervised learning (SSL), new glaucoma diagnostic tools are being developed to enhance accuracy and efficiency, especially as it pertains to interpretation of ODPs^6–11^. These developments may improve accuracy for diagnosis; this is relevant since glaucoma is a silent disease that can remain undiagnosed for years until it progresses to a severe stage. Artificial Intelligence (AI) algorithms, including Convolutional Neural Networks (CNNs) and Vision Transformers, are trained with large datasets of high-quality images. Recent studies examining the relationship between the optic nerve disc and rim for glaucoma screening and progression detection demonstrated the importance of developing these AI-models^9,12–15^. However, most AI applications in glaucoma have been developed on binary classification (e.g., glaucoma vs. non-glaucoma), rather than a more difficult, finer phenotypic differentiation.

In this study, we developed an SSL-based deep learning model (DINO-EYE) with a large dataset of ODPs to predict existing optic disc phenotypes and to identify new variations in patients with POAG. Our model applies the DINO Vision Transformer framework to learn representations of the optic disc morphology. These representations are then used for supervised classification of known phenotypes, as well as unsupervised clustering to explore potential novel phenotypic patterns to better understand the pathophysiologic diversity in glaucoma patients.

## Methods

This retrospective study was conducted using deidentified patient data at the Stein Eye Institute of the University of California, Los Angeles (UCLA); adheres to the tenets of the Declaration of Helsinki; was approved by the UCLA Human Research Protection Program; and conforms to Health Insurance Portability and Accountability Act (HIPAA) policies. Informed consent to participate was waived as approved by the UCLA Bruin Institutional Review Board (IRB).

**Fig. 1:**
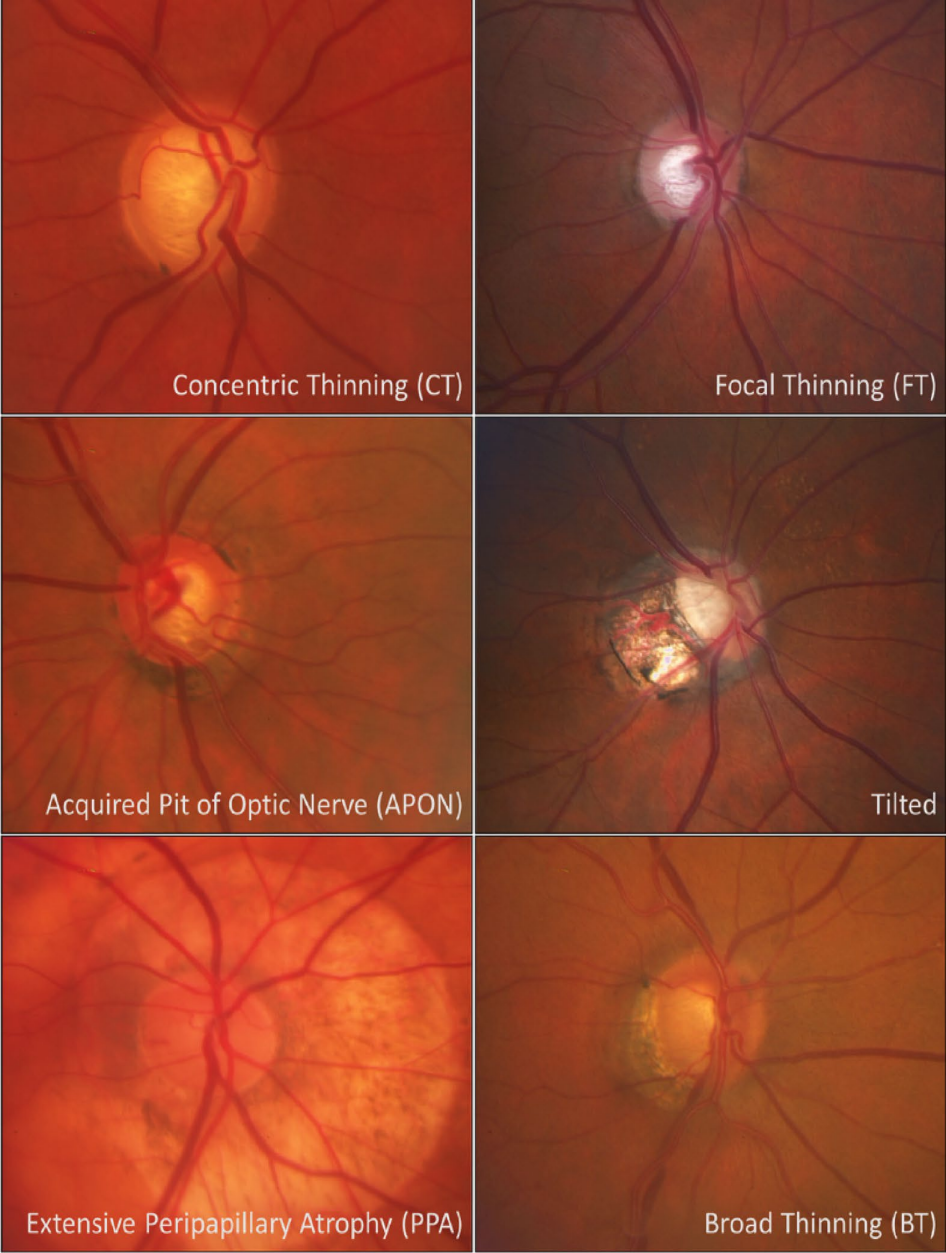
Standard reference photos for phenotypic groups.

Consistent with our prior work^4^, the phenotypes were defined as follows: concentric thinning was defined as a uniform rim thinning. Focal thinning was described as a disc with inferior or superior notching. APON was defined as a deep, localized excavation of the neural rim with sharply localized depression; the affected area is pale, and little or no laminar tissue remains in its depth^5^. Despite being traditionally classified as distinct features, APON and Focal thinning were merged into a single category for analysis in this study due to their significant clinical and pathological overlap. Both phenotypes share a characteristic presentation of localized damage to the neuroretinal rim and are strongly associated with conforming paracentral visual field defects^16^. This high degree of structural and functional similarity justifies their consolidation to create a more robust category for the assessment of localized susceptibility. Additionally, the tilted phenotype was described as vertically tilted which may or may not be associated with peripapillary atrophy (PPA); the extensive PPA phenotype is characterized by a disc with shallow diffuse excavation and 360° of PPA around the disc; PPA is defined by a loss and irregular disruption of the retinal pigment epithelium and of the choriocapillaris in the area surrounding the optic disc^17^. Finally, broad thinning presents with a total loss of disc rim, which is more extensive than a notch, but extends for < 180° of the optic disc circumference (Fig. 1).

A total of 885 color fundus photographs were initially obtained from the UCLA Stein Eye Institute Glaucoma database. Of these, 35 images were excluded due to insufficient quality, yielding a final dataset of 850 ODPs from 850 patients for analysis. The inclusion criteria were eyes with POAG diagnosis, Median Deviation (MD) greater than − 10 dB, and optic disc area between 1 and 3 mm² closest to the date of ODP to avoid eyes with advanced optic nerve damage and loss of phenotypic features.

All color fundus photographs were taken with a Zeiss FF 450plus fundus camera after pupil dilation. As previously published^4^, a web-based interface was created to allow three glaucoma specialists to perform the grading independently. Each image was assigned a single phenotype, corresponding to the most distinctive phenotypic feature chosen for analysis. Agreement was defined as a coincidence choice between at least two of the three graders. Instances of complete disagreement among all three graders were reviewed in a joint meeting. Inter-grader agreement, assessed using unweighted Kappa, is detailed in Table 1.

**Table 1:** Unweighted kappa agreement.

Graders	Agreement %	Kappa	95% CI
1 versus 2	40.00	0.22	0.17 to 0.28
1 versus 3	21.82	− 0.01	− 0.06 to 0.05
2 versus 3	51.69	0.37	0.31 to 0.44

To address class imbalance among phenotypes, we applied offline augmentation of the original images. The original phenotype frequencies and the augmentation ratios were reported in Table 2, where the augmentation ratios are approximately inversely proportional to the frequencies. The augmentations were randomly assigned to be rotation, zooming, and adding Gaussian noise. The following augmentations and parameter ranges were used: (i) Rotation, range 1–10 with a step of 0.5; (ii) Rotation, range − 10 to − 1 with a step of 0.5; (iii) Zoom, range 1.05–1.3 with a step of 0.01; (iv) Gaussian Noise, range 10–100 with a step of 5. To prevent leakage, all augmentations of a given original image were kept in the same split, and phenotype proportions were approximately preserved across splits.

**Table 2:** Phenotype frequency and augmentation ratios of the 850 glaucoma eyes.

Phenotype	Count	Augmentation ratio	Augmented count
APON	25	15	1525
Broad thinning	150	3	1964
Concentric thinning	141	3	1806
Extensive PPA	45	8	1525
Focal thinning	384	1	1908
Tilted	105	4	1765

**Fig. 2:**
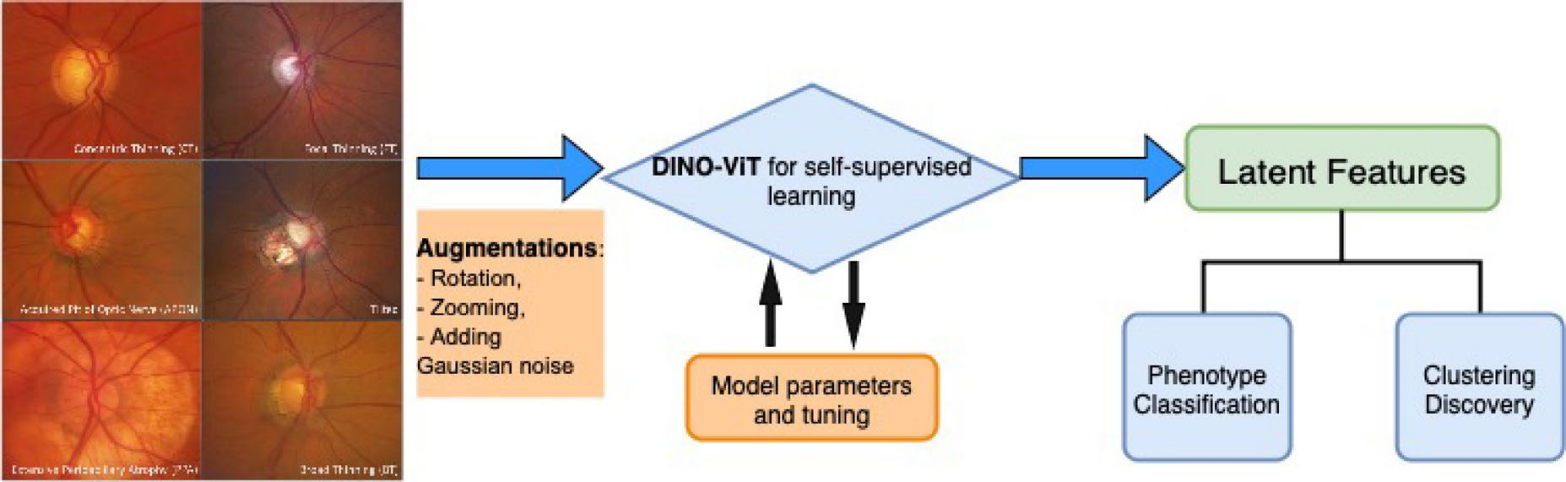
Illustration of the DINO-EYE pipeline: (i) image augmentation; (ii) DINO-ViT model for self-supervised learning; (iii) phenotype classification and clustering on the learned latent features.

**Fig. 3:**
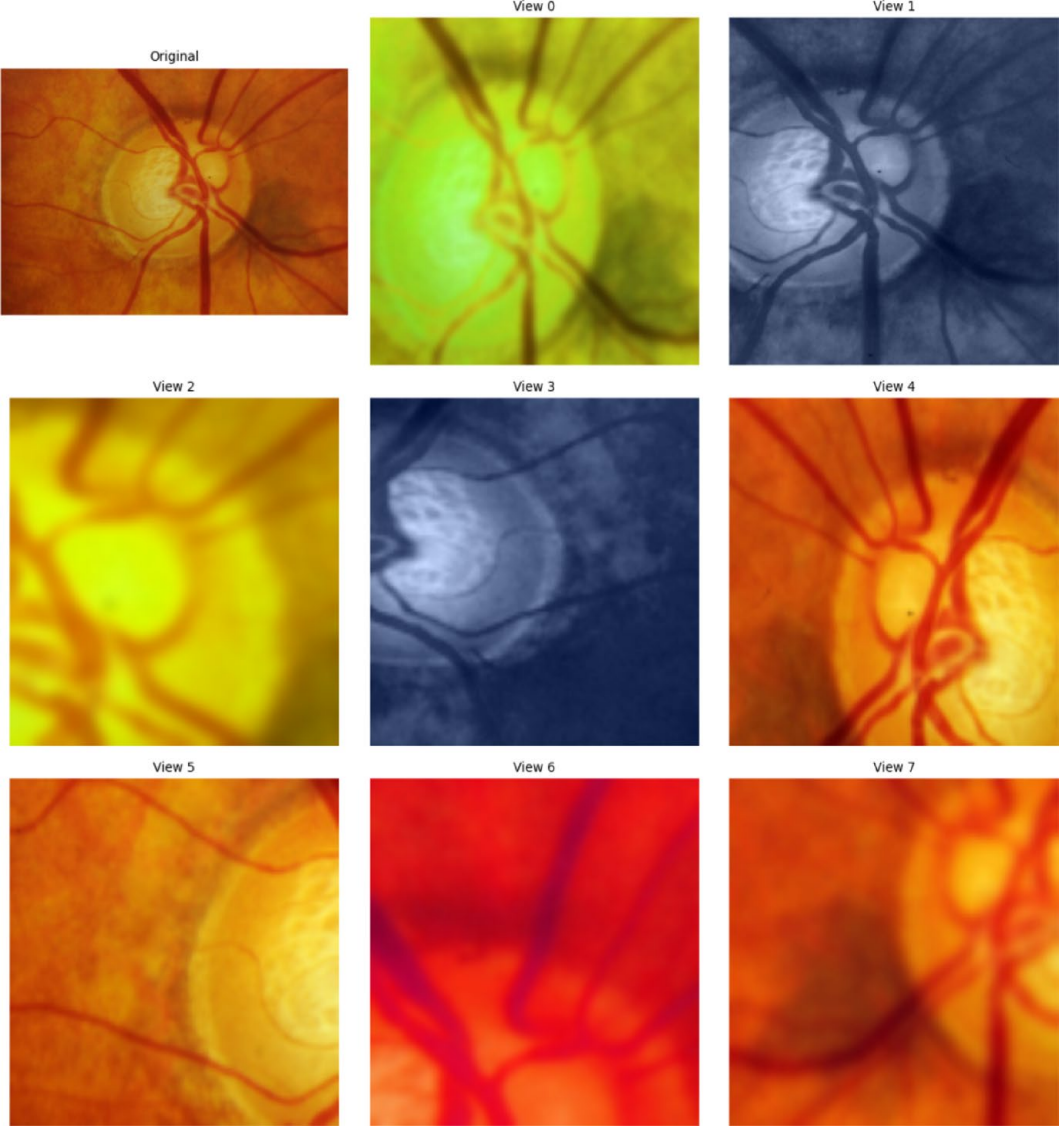
8-view panels of the same glaucoma image by DINO backbone model, including 2 global crops and 6 local crops that focus on different parts of the image. The original image is in the top left corner.

SSL models were developed to extract high level features/representations of the original images, which can be used for downstream tasks, like predicting phenotypes of interest. We used the DINO (self-distillation with no labels) Vision Transformer^18^ as the backbone model (Fig. 2), which uses a teacher–student (self-distillation) framework and generates different views of the same input image with augmentations (see also BYOL^19^ to learn the common and distinct features from these views (Fig. 3).

Then, the outputs of the DINO model, 2048-dimensional feature vectors, serve as high-level latent representations of potential clinical characteristics of the original images. Subsequent analyses were performed on these high-level features learned from the DINO model. Specifically, the extracted features were used as input for supervised phenotype classification. Two ensemble-based classifiers, Random Forest and XGBoost, were evaluated on the 2,209-image held-out test set. A grid search of hyperparameters was performed for both methods. The parameter space for random forest was: number of trees in (200, 500, 800); max tree depth in (3, 5, 7); min samples per leaf in (1, 3, 5). For XGBoost, the grid included: number of boosting trees in (50, 100), max depth in (5, 7), learning rate in (0.01, 0.05), subsample fraction in (0.6, 0.8), fraction of features in (0.6, 0.8), and gamma in (0.1, 0.3). Within this subset we performed the same stratified 80/20 split as before (preserving phenotype proportions). Models were trained on the training partition and accuracy was reported on the test partition.

Lastly, to gain insight into the regions of the images that most strongly influenced model predictions, we generated class-specific attention maps. These visualizations allowed for qualitative evaluation of model focus across different phenotypes. The attention maps can potentially highlight clinically meaningful features, offering reassurance that the DINO model’s internal representations aligned with expert-driven diagnostic criteria.

For exploratory purposes, we applied unsupervised clustering to the 2048-dimensional latent features. Because the imbalanced true phenotypes, like APON and extensive PPA have much lower frequency than others, we fixed the number of clusters at five to facilitate comparison with the clinical phenotypes and to avoid over-partitioning. Two clustering methods were performed, including K-means and Agglomerative Clustering (Agglo). Three experienced clinicians reviewed representative samples from each cluster to assess whether novel phenotypic patterns could be identified. UMAP (Uniform Manifold Approximation and Projection) was used to reduce feature dimensionality for visualization, highlighting several coherent clusters. We defer the results to Supplementary Materials (Figs. S1 and S2).

## Results

### Phenotype demographics and dataset augmentation

Table 3 details the frequency distribution of the demographic characteristics among the phenotypes. The overall study population was predominantly female (58.2%). The largest reported Racial group was Caucasian (57.6%). The Tilted phenotype contained the highest proportion of Asian participants (41.9%) and the lowest median age (65 years). Regarding gender distribution within the phenotypes, the broad thinning group showed the greatest proportion of females (62.7%), while the concentric thinning phenotype had the highest proportion of males (54.6%). The total number of augmented images is *N* = 10,493, and the augmented sample sizes for each phenotype are shown in the last column of Table 2. We split the 10,493 augmented images into an 80/20 train/test set (8,284/2,209).

**Table 3:** Demographic distribution across the different phenotypes.

Phenotype	*N*	Gender		Race					Age
		Male (%)	Female (%)	Caucasian (%)	Asian (%)	African descent (%)	Hispanic (%)	Other (%)	Median (IQR)
All	850	355 (41.8)	495 (58.2)	490 (57.6)	136 (16.0)	81 (9.5)	37 (4.4)	106 (12.5)	72 (15)
Concentric thinning	141	77 54.6	64 (45.4)	80 (56.7)	12 (8.5)	16 (11.3)	9 (6.4)	24 (17.0)	72 (14)
Focal thinning	384	148 (38.5)	236 (61.5)	232 (60.4)	51 (13.3)	37 (9.6)	16 (4.2)	48 (12.5)	72 (15)
APON	25	10 (40.0)	15 (60.0)	17 (68.0)	3 (12.0)	3 (12.0)	0 (0.0)	2 (8.0)	68 (8)
Tilted	105	44 (41.9)	61 (58.1)	41 (39.0)	44 (41.9)	7 (6.7)	3 (2.9)	10 (9.5)	65 (14)
Extensive PPA	45	20 (44.4)	25 (55.6)	32 (71.1)	4 (8.9)	2 (4.4)	2 (4.4)	5 (11.1)	74 (15)
Broad thinning	150	56 (37.3)	94 (62.7)	88 (58.7)	22 (14.7)	16 (10.7)	7 (4.7)	17 (11.3)	72 (13)

## Model training and feature extraction

We used a batch size of 32, the pretrained “dino_vits16” as the backbone model with token/hidden dimension 384 (i.e. the embedding dimension of each 16 × 16 patch/CLS token, distinct from the SSL projection head with 2048 latent feature dimension), a learning rate of 0.001, a maximum epoch of 250, and an early stopping criterion of patience = 50. The DINO-EYE SSL model successfully learned 2048 features from the original input images.

## Phenotype classification using SSL features

**Fig. 4:**
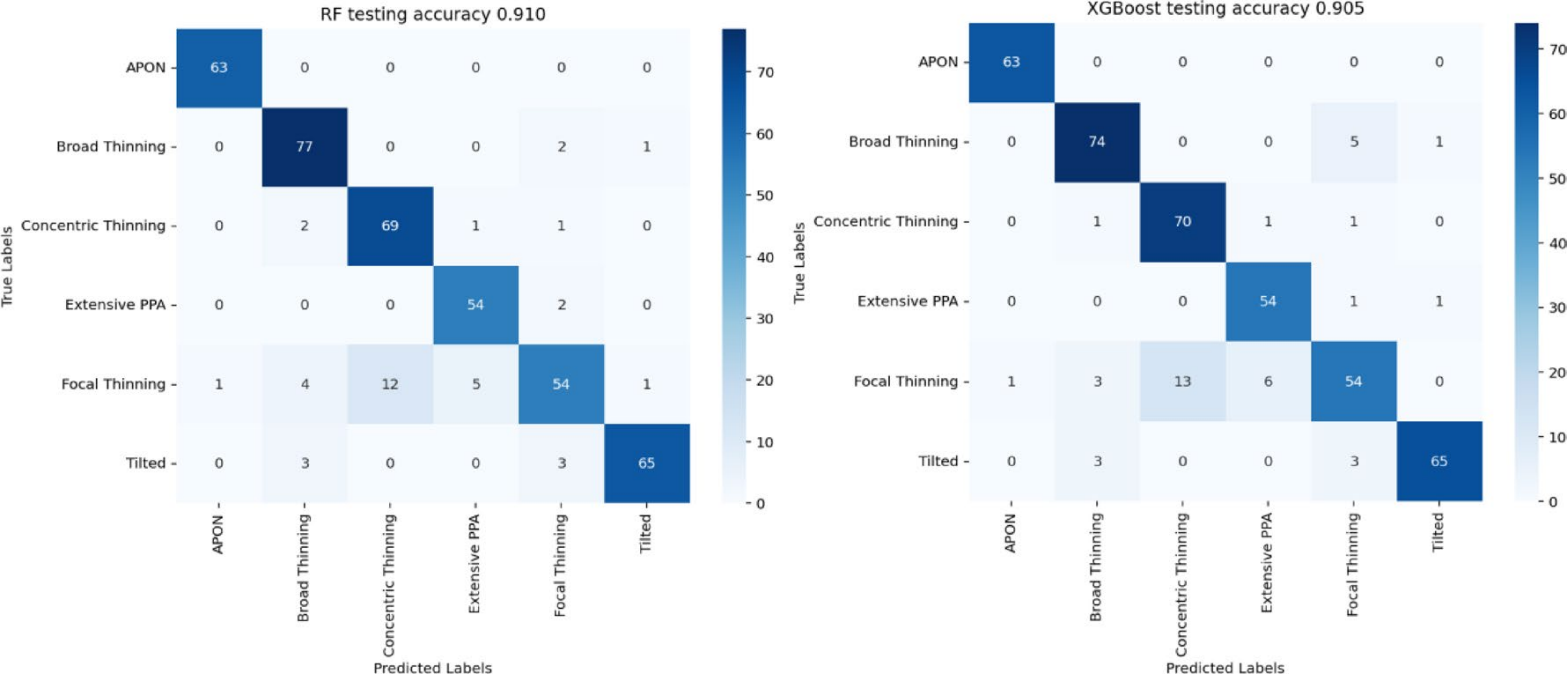
Phenotype classification using 2048 SSL features and 6 phenotypes on the held-out augmented dataset. Left: random forest; Right: XGBoost. Confusion matrices of 420 test images are reported.

**Fig. 5:**
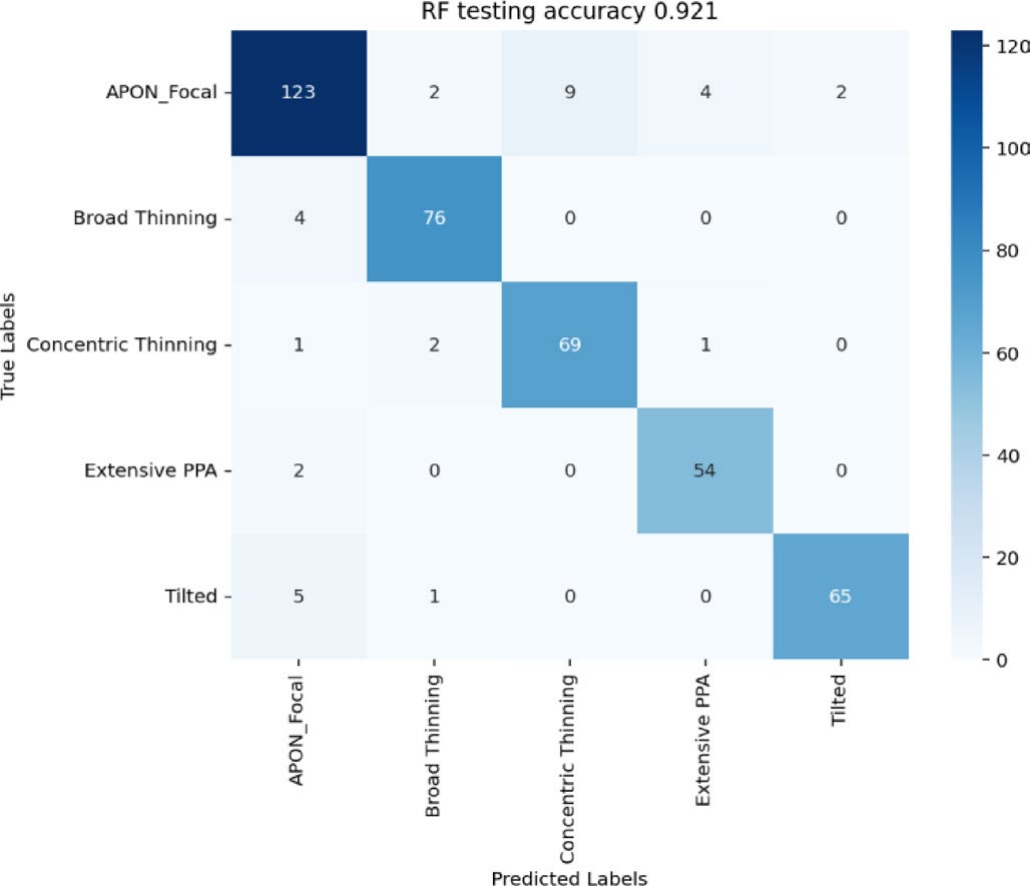
Phenotype classification using 2048 SSL features and 5 phenotypes (APON and focal thinning combined).

We further applied random forest and XGboost for phenotype classification using these features, on the held out augmented dataset (*N* = 2,209). We achieved an accuracy of 91% to identify the true phenotypes on the test set with random forest, and accuracy of 90.5% with XGboost (Fig. 4). Since APON and focal thinning are two clinically similar phenotypes, we combined them into one and repeated the classifications. This classification improved to 92.1% accuracy with random forest (Fig. 5).

## Attention map interpretability

**Fig. 6:**
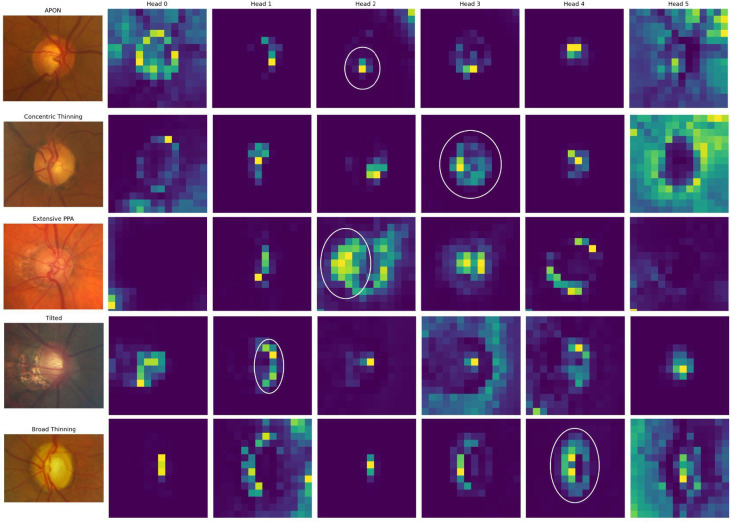
Attention head maps for each phenotype. The clinical characteristic pattern is circled in white.

We created attention maps of the images by each phenotype to better understand what features the DINO model learned that are clinically meaningful and can be matched to the phenotype (Fig. 6). Overall, attention heads 0 and 5 do not appear to be related to any phenotypes. In the given examples, attention head 1 reveals feature related to the tilted phenotype; attention head 2 is related to extensive peripapillary atrophy; attention head 3 is related to concentric thinning; and attention head 4 is related to broad thinning.

## Comparison with retfound model

Lastly, we applied the RETFound^19^ pretrained model to our data for comparison and external validation. The pretrained RETFound model was fine-tuned on our data and learned 1024 latent features for subsequent analyses. We adapted the code provided by the RETFound authors, specifically their main_finetune python code and input size 224, patch size 16, learning rate 0.005 with layer_decay 0.65, and 100 epochs for the fine tuning. Phenotype classification with random forest was performed with these latent features on the same 2,209 held-out augmentation samples. The best performance achieved 0.89 test accuracy, which was slightly worse than the DINO-EYE model.

## Discussion

In this study, we developed the DINO-EYE SSL model that can identify different phenotypes in patients with POAG using a unique dataset of 850 ODPs. Building on prior classifications by Nicolela and Drance^3^, and Grassi et al.^4^, our model addresses the limitations posed by inter-observer variability and subjective bias in phenotype classification.

Our work is situated within the developing literature of employing SSL for enhanced diagnostic and interpretability in ophthalmology, providing visual and textual explanations. While a recent study^20^ has advanced to explore multimodal interpretability, combining visual explanations (e.g., heatmaps) with textual output (e.g., AI chatbots), our focus is on generating accurate and clinically validated visual interpretations. We use attention maps of the latent features, to ensure the model’s decision-making is transparent and consistent with expert criteria. Furthermore, we will explore powerful tools like LLMs (Large Language Models) and AI chatbots in future work.

The SSL machine for identification of the different phenotypes offers an alternative to reduce human error in the classification. The RETFound model^21^, a SSL foundation model that was trained with a large number of images (1.6 million of unlabelled data), was also applied to our data to learn generalizable representations from retinal images. However, this more general model does not show superior performance on our dataset, suggesting a limitation in the generalizability of such models. Notably, we highlight the interpretability of our model through attention maps, key to clinician relevance and common usage.

The proposed DINO-EYE achieves the best performance with an accuracy of 92.1% in identifying the different phenotypes, based on 850 images from a previous study. In comparison, Benton Chuter et al.^23^, offered an AI model using RETFound to classify ODP in “Glaucomatous” or “Non-glaucomatous”. With fine-tuning on the same dataset, RETFound only achieved an accuracy of 89% on the same phenotype classification task. Compared with RETFound—which was pretrained on broad retinal datasets and only lightly fine-tuned on our cohort—DINO-EYE is trained end-to-end on in-domain ODPs, yielding representations tailored to POAG phenotypes. This in-domain SSL reduces domain shift and captures phenotype-specific cues, translating into higher accuracy on our dataset. While foundation models like RETFound offer flexibility and external generalizability, our results show that task-specific training on a moderate, well-curated dataset can outperform generic pretraining for phenotype classification.

Despite promising classification performance, our attempt to identify novel phenotypes through unsupervised clustering did not yield clearly new categories. Nevertheless, the coherent groupings observed suggest that further exploration with larger and more diverse datasets could refine existing subclassifications or reveal subtle phenotypic variants.

Key limitations should be acknowledged. First, our dataset, although augmented, originated from a single clinical center and included only 850 original images, which may limit the model’s generalizability. However, this is mitigated by the novelty of our study. Multi-center validation with demographically diverse populations is essential to confirm robustness. Second, the phenotype labels used in supervised classification were derived from expert grading, which can be inherently subjective. Additionally, although the model’s attention maps provide interpretability, the risk of “data fishing” remains. Future directions include: (i) expanding the dataset with other collections worldwide; (ii) experimenting with other augmentation techniques to address imbalance classes more effectively; (iii) incorporate feature selection of SSL latent features that are specific to clinical outcomes, such as progression analysis or treatment response. We see this model being used for decision support in the real-world, providing an unbiased opinion to non-glaucoma specialists or for training purposes.

Our study is among the few that apply SSL to the more challenging problem of glaucoma phenotyping. Other recent SSL applications in ophthalmology have largely focused on disease detection or segmentation tasks^23–25^. Related works include PaRCL^26^, a self-supervised contrastive learning method for glaucoma classification using fundus images; a semi-supervised learning^27^ method for retinal fundus image segmentation via self-training based on the MR-Net; One-Vote Veto (OVV) self-training^28^, a semi-supervised learning strategy for glaucoma diagnosis using unlabeled fundus images. By contrast, SSL methods on phenotypes are scarce. Our focus on phenotype-level classification opens pathways for individualized treatment planning and provides the visual representation necessary for future incorporation of AI chatbots with multimodal AI systems in glaucoma management.

## Conclusion

We developed DINO-EYE, a self-supervised learning framework that successfully extracted meaningful features from ODPs. We used the SSL feature to accurately classify clinically assigned optic disc phenotypes in POAG. The DINO-EYE model achieved phenotype classification accuracy of 90% or higher. The model outperforms the existing state-of-art approach RETFound on the same dataset and has the potential to reduce clinician subjectivity in phenotype classification. However, no new phenotypes were confirmed by clinicians in this study. Further validation and scaling are warranted for clinical deployment and discovery of novel disease subtypes.

## Supplementary Information

Below is the link to the electronic supplementary material.

Supplementary Material 1.

## Data Availability

The datasets generated during and/or analysed during the current study are not publicly available due to patient privacy. For more information, please contact the corresponding author, Joseph Caprioli, Jules Stein Eye Institute, Los Angeles, CA 90095, USA; Caprioli@jsei.ucla.edu.
