# Supplementary material for: DINO-EYE: self-supervised learning for identification of different optic disc phenotypes in primary open angle glaucoma
Source: Sci Rep. 2026 Jan 10;16:3206. doi: 10.1038/s41598-025-33140-1 (PMC12830587; doi:10.1038/s41598-025-33140-1)
Supplement: Supplementary file 1 — Supplementary Material 1. [file 41598_2025_33140_MOESM1_ESM.docx]

**Clustering for Different Phenotypes**

We conducted an unsupervised analysis on the learned latent features of the SSL to assess the stability and consistency of the phenotypic grouping. To ensure a comprehensive exploration of clustering behavior, we employed two different clustering algorithms: K-means and Agglomerative Clustering. Agglomerative Clustering produced the more clinically coherent groupings and demonstrated the highest alignment with the clinically assigned phenotype labels. Several clusters showed high internal consistency, particularly for concentric thinning and extensive PPA. However, considerable overlap was observed among other phenotypes, suggesting areas for re-evaluation of phenotype boundaries.

The resultant clusters were examined by a panel of three glaucoma-trained ophthalmologists to determine if the groupings corresponded to any previously uncharacterized or "novel" phenotypes. Due to the high degree of inter-cluster overlap with existing clinical labels, no distinct, novel clinical phenotypes were confirmed by the expert panel.

| Cluster | 0 | 1 | 2 | 3 | 4 |
| --- | --- | --- | --- | --- | --- |
| APON | 5 (20%) | 3 (12%) | 6 (24%) | 3 (12%) | 8 (32%) |
| Broad Thinnging | 40 (26.7.%) | 24 (16%) | 42 (28%) | 19 (12.7%) | 25 (16.7%) |
| Concentric Thinning | 20 (14.2%) | 25 (17.7%) | 43 (30.5%) | 21 (14.9%) | 32 (22.7%) |
| Extensive PPA | 22 (48.9%) | 9 (20%) | 12 (26.7%) | 2 (4.4%) | 0 (0%) |
| Focal Thinning | 73 (19%) | 64 (16.7%) | 117 (30.5%) | 61 (15.9%) | 69 (18%) |
| Tilted | 18 (17.1%) | 20 (19%) | 24 (22.9%) | 16 (15.2%) | 27 (25.7%) |
| APON + FT | 78 (19.1%) | 67 (16.4%) | 123 (30.1%) | 64 (15.6%) | 77 (18.8%) |

Table S1. Distribution of optic disc phenotypes across the five clusters identified by K-means clustering. Numbers indicate the count and percentage of eyes belonging to each phenotype within each cluster.

| Cluster | 0 | 1 | 2 | 3 | 4 |
| --- | --- | --- | --- | --- | --- |
| APON | 6 (24%) | 8 (32%) | 5 (20%) | 1 (4%) | 5 (20%) |
| Broad Thinnging | 35 (23.3%) | 46 (30.7%) | 34 (22.7%) | 25 (16.7%) | 10 (6.7%) |
| Concentric Thinning | **50 (35.5%)** | **53 (37.6%)** | 16 (11.3%) | 11.7 (7.8%) | 11 (7.8%) |
| Extensive PPA | 4 (8.9%) | 6 (13.3%) | **24 (53.3%)** | 3 (6.7%) | 8 (17.8%) |
| Focal Thinning | 116 (30.2%) | 102 (26.6%) | 70 (18.2%) | 50 (13%) | 46 (12%) |
| Tilted | **39 (37.1%)** | 32 (30.5%) | 14 (13.3%) | 6 (5.7%) | 14 (13.3%) |
| APON + FT | 122 (29.8%) | 110 (26.9%) | 75 (18.3%) | 51 (12.5%) | 51 (12.5%) |

Table S2. Distribution of optic disc phenotypes across the five clusters identified by Agglomerative clustering. Numbers indicate the count and percentage of eyes belonging to each phenotype within each cluster.


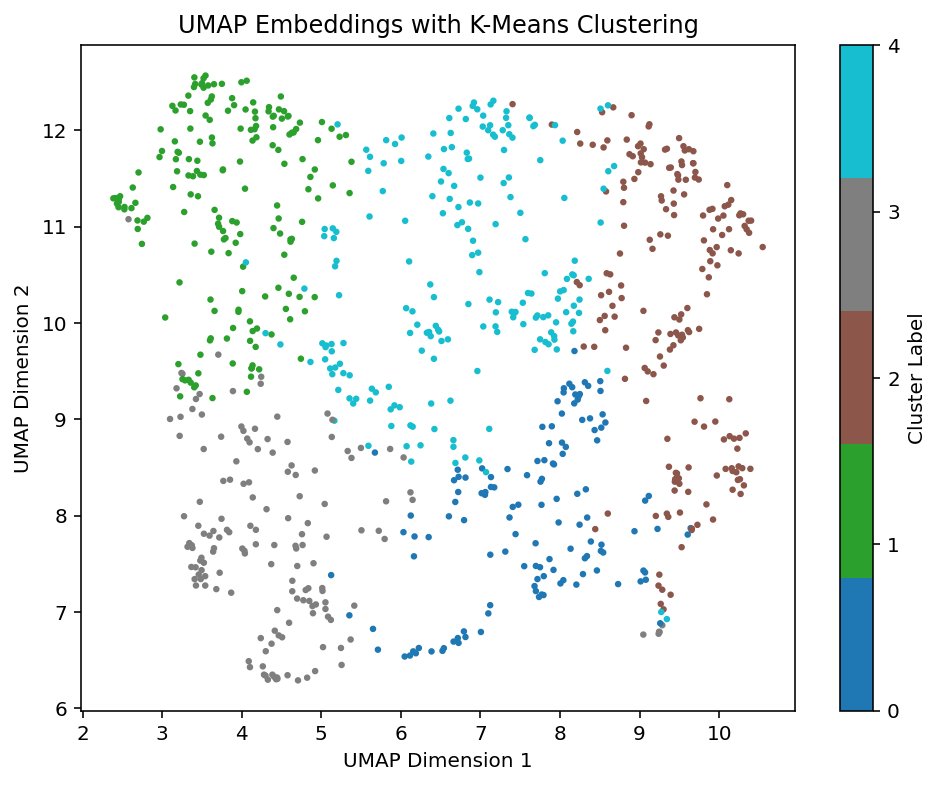


Figure S1. UMAP visualization of DINO latent features. Clustering was based on the K-means Clustering method.


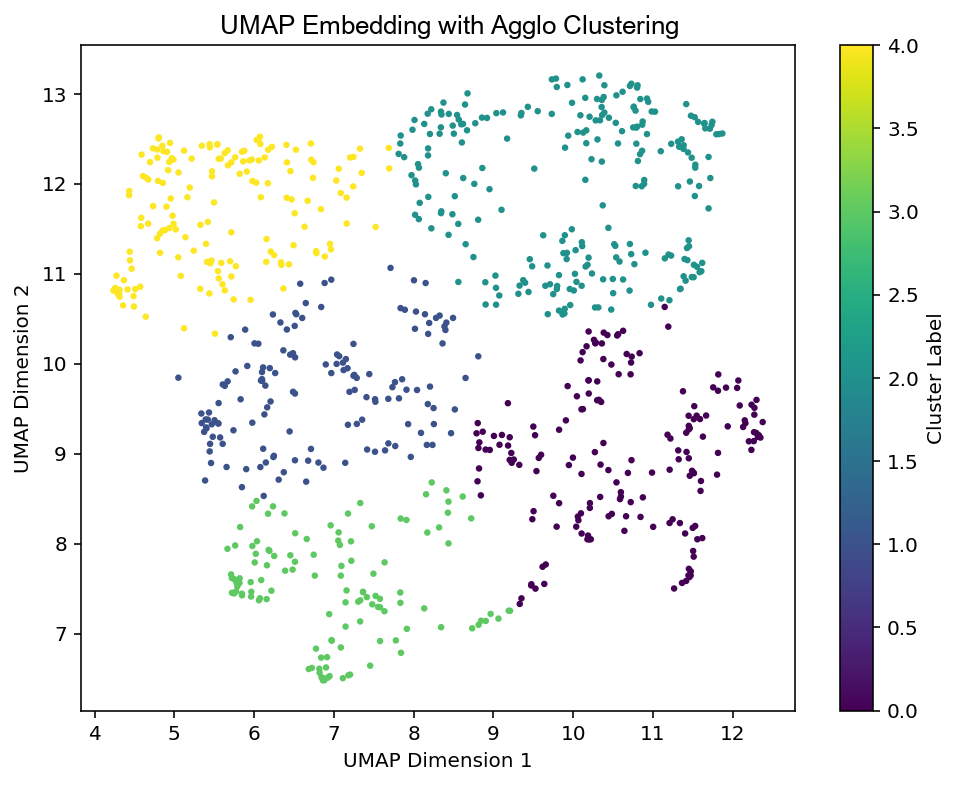
Figure S2. UMAP visualization of DINO latent features. Clustering was based on the Agglomerative clustering method.
